# Alterations of Neocortical Pyramidal Neurons: Turning Points in the Genesis of Mental Retardation

**DOI:** 10.3389/fped.2014.00086

**Published:** 2014-08-11

**Authors:** Alberto Granato, Andrea De Giorgio

**Affiliations:** ^1^Department of Psychology, Catholic University, Milan, Italy

**Keywords:** apoptosis, dendrites, calcium spikes, fetal alcohol spectrum disorders, dendritic spines

## Abstract

Pyramidal neurons (PNs) represent the majority of neocortical cells and their involvement in cognitive functions is decisive. Therefore, they are the most obvious target of developmental disorders characterized by mental retardation. Genetic and non-genetic forms of intellectual disability share a few basic pathogenetic signatures that result in the anomalous function of PNs. Here, we review the key mechanisms impairing these neurons and their participation in the cortical network, with special focus on experimental models of fetal exposure to alcohol. Due to the heterogeneity of PNs, some alterations affect selectively a given cell population, which may also differ depending on the considered pathology. These specific features open new possibilities for the interpretation of cognitive defects observed in mental retardation syndromes, as well as for novel therapeutic interventions.

Santiago Ramón y Cajal referred to the neocortical pyramidal neuron (PN) as “La noble y enigmática célula del pensamiento” (the noble and enigmatic cell of thought) (1). These glutamatergic, excitatory neurons represent the vast majority of neocortical cells (about 80–90%), the remaining being constituted by GABAergic, inhibitory interneurons. Surprisingly and contrary to what one may expect, cortical interneurons, though minor in number, are characterized by a great variety of anatomical features, electrophysiological properties, and synaptic attributes [see Ref. (2) for review]. Conversely, PNs are often conceived as a rather homogeneous population. However, the principal neurons of the cerebral cortex are far from being identical to each other, since they show both evident and more subtle differences (Figure 1). In the present mini-review, we will first provide some examples of how PNs represent a heterogeneous population. Then, while it is quite obvious that developmental disorders associated with mental retardation (MR) target the main structure involved in cognitive functions (i.e., the cerebral cortex) and its majority neurons, we try to answer the question whether given subpopulations or functional features of PNs are preferentially affected. We focus mainly on the effects of fetal exposure to alcohol (see Figure 2), highlighting analogies and differences with other developmental disorders associated with MR.

**Figure 1:**
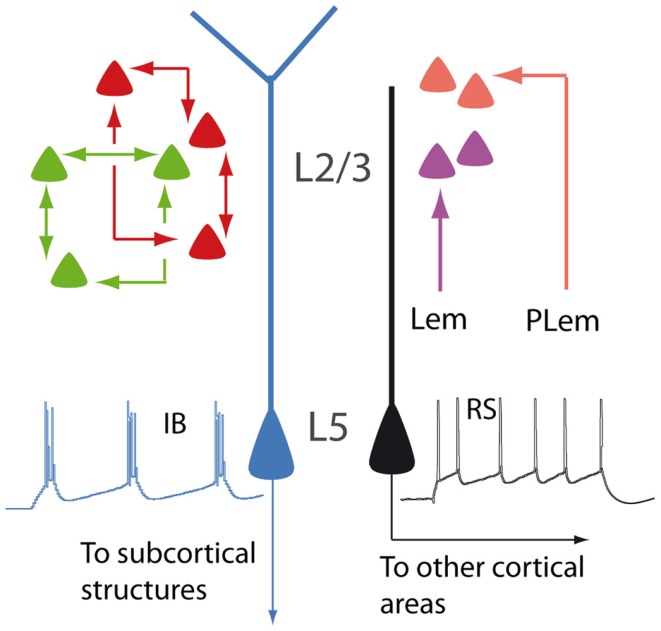
**Schematic illustration showing different types of PNs**. In layer 2/3, neurons are interconnected to form distinct subnetworks (green and red cells). In the barrel cortex, lemniscal (Lem) and paralemniscal (PLem) afferents target different subpopulations (purple and orange cells). In layer 5, regular spiking PNs (RS, black) and intrinsically bursting PNs (IB, blue) display different dendritic morphologies and different projections.

**Figure 2:**
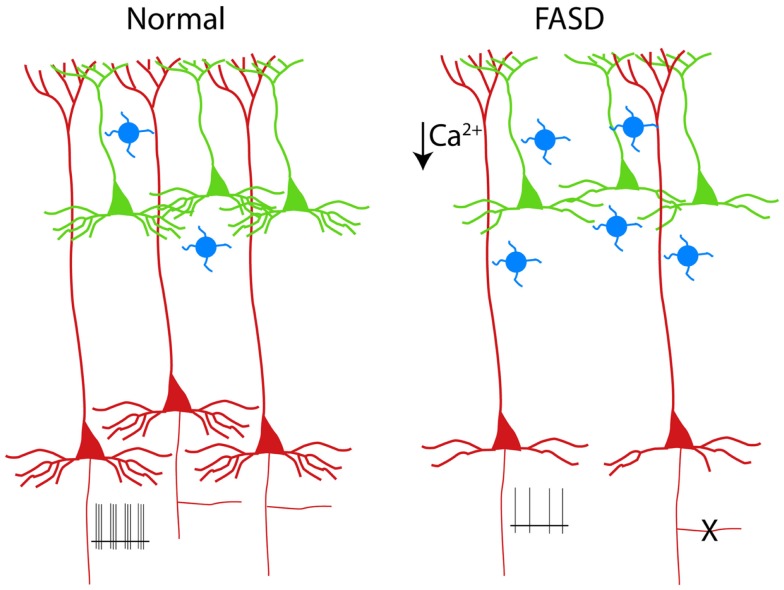
**Schematic drawing of PNs in a normal cortical column (left)**. The alterations of PNs observed in experimental models of FASD are shown on the right (3–6). Supragranular and infragranular PNs are shown in green and red, respectively. Note the reduced population of L5 PNs (or the pruning of their axon collateral, **X**) and the simplification of the basal (but not apical) dendrites. The hypoexcitability of L5 PNs (shown as reduced number of spikes at the axon level) is a consequence of reduced dendritic calcium spikes (5). The increased number of calretinin interneurons (blue cells) is also shown (7).

## Heterogeneity of PNs

The difference among PNs is already apparent at a first glance of histological sections and is related to their radial position within the six-layered neocortical sheet. Besides the obvious morphological difference (short vs long apical dendrites), supragranular (layer 2/3; L2/3) and infragranular (layer 5; L5) PNs participate differently to the flow of information in the canonical microcircuit of the cortical column (8). Differences between supra- and infragranular layers can be observed also when looking at the more subtle, intrinsic electrophysiological properties. For instance, L2/3 neurons display less hyperpolarization-activated currents (I_h_), compared to L5 neurons (9).

The analysis of the fine columnar connections makes it possible to further distinguish subpopulations within L2/3 neurons. In the barrel cortex, for instance, lemniscal and paralemniscal afferents target PNs located at different depths in the supragranular layers (10). In the rodent visual cortex, L2/3 neurons are selectively interconnected to form fine-scale, distinct subnetworks (11).

Layer 5 PNs can be also further subdivided into subsets featuring discrete properties. Based on morphology, electrophysiology, and functional connectivity, L5 PNs are classified into intrinsically bursting and regular spiking. The former have the tendency of firing bursts of action potentials in response to steps of depolarizing current, usually display a prominent apical tuft in layer 1 (thick-tufted), and project to subcortical targets. The latter fire trains of action potentials with constant interspike intervals, have a slender apical dendrite, and project mainly to other cortical areas (12–14). Within layer 5, PNs belonging to the sparse L5a and the densely populated L5b are also clearly distinguishable, according to differences concerning functional and connectional properties (15, 16). Even when L5 PNs project to the same subcortical target, they may be involved in different functional circuits, as it has been proposed for cortico-striatal neurons sustaining the direct and indirect pathways of the basal ganglia, respectively [(17); but see Ref. (18)]. The parcelation of PNs according to their radial distribution is further complicated by the heterogeneous population of layer 6 neurons (19). The apical dendrites of these cells are unusual, as they, unlike those of other PNs, do not reach superficial layers, although sharing many electrophysiological properties with other neocortical PNs (20).

If the uneven properties of PNs along the radial cortical dimension reflect the structure-function relationship within the column microcircuit, equally outstanding is the diversity along the tangential dimension. In this regard, the complexity of the dendritic tree increases as one moves from primary sensory to higher order areas, reaching the most complex pattern in the prefrontal cortex (21). Further, the prefrontal cortex contains a large number of unusual PNs, which display an early bifurcation of the apical dendrite, whose total length is therefore susbstantially increased (22).

We have briefly outlined the laminar and regional heterogeneity of PNs. However, the reader should bear in mind that, even if neocortical PNs were homogeneous across cortical areas and layers, nonetheless each of them would represent the most complex neuron of the mammalian brain. Let us consider, for example, the L5 PN. Its apical dendrite extends through most of cortical thickness and is thus ideally suited for translaminar integration. In addition, the long, apparently homogeneous dendritic arbor of these neurons features specific functional properties: basal dendrites and the apical tufts are dominated by NMDA spikes, while Ca^2+^ spikes sustained by voltage-gated channels prevail in the distal apical trunk (23). Finally, dendritic, axon, and somatic domains of L5 PNs are targeted by different types of inhibitory interneurons (24). In summary, even the single PN is a complex world itself, able to integrate feedforward ascending input and feedback connections to generate the cognitive performance (25).

## Apoptosis

Early exposure to alcohol, whose effects are globally referred to as fetal alcohol spectrum disorders (FASD), are well known causes of mental retardation. There are manifold factors involved in the neurodevelopmental toxicity of ethanol, which is critically dependent on the dose and time of exposure [see Ref. (26), for review]. Experimental models of FASD allow a tight control of alcohol exposure and help to dissect out the mechanisms operant at different developmental stages. When rodents are exposed during prenatal life, alcohol is more likely to interfer with the proliferation of neuron precursors and/or with the migration of cortical cells (27, 28). By contrast, when rodents are given alcohol during the first two postnatal weeks [corresponding to the third trimester of gestation in humans, see Ref. (29)], a massive apoptosis occurs in several brain structures, including the cerebral cortex (30). The third trimester equivalent is characterized by intense synaptogenesis and the alcohol-induced apoptosis is thought to be caused by the simultaneous blockade of NMDA receptors and activation of GABA receptors (31). The apoptosis observed in the neocortex after postnatal alcohol exposure in rodents seems to affect mainly infragranular PNs, as demonstrated by the selective presence of molecular markers of apoptotic susceptibility, such as caspase 3 and the low-affinity neurotrophin receptor (p75 NTR), in L5 cells [(3, 32); see Figure 2]. The prevailing involvement of infragranular PNs is also suggested by the increased ratio between supragranular and infragranular PNs sustaining the cortico-cortical associative projections (4). Notably, the vulnerability of these neurons to apoptosis outlasts the alcohol exposure, since an increased immunoreactivity for p75 NTR is observed several days after withdrawal (3). In a different experimental model of MR, reproducing the congenital hypothyroidism, the increased apoptosis is associated to upregulation of p75 NTR (33). In this case, however, the apoptotic cells are confined to supragranular instead of infragranular layers (33).

The unbalanced weights of supra- and infragranular layers, as observed in different types of MR, can yield important functional consequences. For instance, sensory and memory processing carried out by the same cortical area are mediated by opposite flows of interlaminar signals [supragranular → infragranular and infragranular → supragranular, respectively; see Ref. (34)].

It is worth noting here that experimental models mimicking other types of MR are characterized by a reduced rate of naturally occurring cell death, rather than by increased apoptosis. This is the case for FMR1 mutants (reproducing the fragile X syndrome) and for the Rett syndrome as well (35, 36). Thus, it appears that both the excess of apoptosis and the lack of programed cell death can equally lead to an impairment of the cortical network and to cognitive defects.

## Dendrites and Connectivity

The dendritic tree of PNs, with its long and extensively ramified branches, must be considered the main computational device of the neocortex (37). Therefore, it is not surprising that dendritic alterations are recognized as the key anatomical counterpart of MR (38). In experimental models of FASD based on early postnatal exposure, the basal dendritic arbor of PNs is more affected, as compared to the apical dendrite [(4, 5); see Figure 2]. Basal dendrites of L2/3 associative PNs in alcohol-treated rats display fewer dendritic branches than in controls, suggesting a defect of branching rather than of terminal dendrite elongation (6). This dissociation can be justified by the different molecular machinery involved in the two distinct phenomena of branching and terminal elongation (39). In the Ts65Dn mouse model of Down syndrome, the basal dendrites of L2/3 PNs, similarly to what observed in FASD, display a reduced complexity of the branching pattern (40). However, in humans affected by Down syndrome, dendritic alterations follow a complex temporal sequence, resulting in a simplification that is more dramatic for apical dendrites (41). A Golgi study by Armstrong and coworkers (42) provides a direct comparison between the dendritic anomalies of Rett and Down syndrome, pointing out that basal dendrites of the frontal cortex in individuals affected by Rett syndrome are strongly impaired both in supra- and infragranular layers, while apical dendrites are affected only in supragranular layers. In experimental models of early-onset hypothyroidism, finally, both apical and basal dendrites of PNs appear to be strongly reduced (43).

Understanding which dendritic domain of PNs is preferentially targeted by disorders associated to MR is not trivial. In fact, basal and apical dendrites not only display different branching patterns, but are also characterized by different functional properties and are likely to play distinctive roles in the cortical network. Apical dendrites receive long-range feedback input from higher order cortical areas (44) and display both Ca^2+^ and NMDA spikes, whereas basal dendrites support only NMDA spikes (45).

Another central issue concerning the relationship between dendrites and MR is represented by the density and distribution of dendritic spines. Most inputs synapsing upon PNs occur on these small protrusions, which are essential for the linear summation of excitatory potentials (46). Almost all disorders associated with MR feature alterations of the number and/or shape of dendritic spines (38). Although a systematic review of dendritic spine anomalies is beyond the aim of the present paper, it is worth mentioning that both a decreased and an increased number of spines can lead to MR. While a reduction of dendritic spines has been observed in experimental models of FASD [e.g., Ref. (47)], their number is significantly higher in fragile X mice (48). Once again, as already pointed out for neuronal populations (see above), also the dendritic spines seem to ensure the good functioning of PNs only if they reach an optimal number. Fewer or more spines, conversely, can equally lead to defective function.

Since each spine is thought to represent the site of at least one synaptic contact, quantitative and/or qualitative spine anomalies are likely to reflect alterations of cortical connectivity. Thus, dendritic alterations can be accompanied by a defect of axon outgrowth or pruning, as demonstrated for early exposure to ethanol (49, 50), for mouse models of Rett syndrome (51), and fragile X syndrome (52). The obvious consequence is a modified intracolumnar (53) and long-range connectivity (4). The main alterations observed in experimental models of MR are summarized in Table S1 in Supplementary Material.

## PN Excitability

The excitability of PNs (i.e., the ability of generating action potentials in response to depolarizing current) depends primarily on the intrinsic membrane properties and, to some extent, on the cited complexity of the dendritic tree. In fact, PN dendrites are not merely passive cables, but they are also endowed with a great variety of active conductances (54). Dendritic voltage-gated channels, in turn, can influence the axo-somatic firing pattern of PNs (55). We have demonstrated that exposure to ethanol during the third trimester equivalent leads to a long-lasting reduction of excitability in L5 PNs (5). Such an impairment represents the consequence of decreased spikes in the Ca^2+^ electrogenesis zone of the apical dendrite. These spikes are usually mediated by voltage-gated Ca^2+^ channels and are accompanied by their somatic counterpart, consisting of a prominent afterdepolarization. Interestingly and in agreement with our observation, Sánchez-Alonso et al. (56), in a mouse model of congenital hypothyroidism, noted that hippocampal PNs showed a decreased afterdepolarization.

An alteration of Ca^2+^ signaling has been also observed in experimental models of fragile X syndrome (57). This condition, however, is rather characterized by hyperexcitability (58). Besides affecting the neuron excitability, the unreliability of Ca^2+^ signals can alter the neural plasticity, as consistently observed in experimental models of MR (57, 59, 60).

## Concluding Remarks

It seems pretty clear that the different etiological factors involved in different types of MR converge upon a few basic mechanisms, regardless of the vast variety of molecular pathways leading to such disturbances. Most of these alterations impair the functional properties of the major cell type of the neocortex, i.e., the PN. Here, we have briefly described some of the main mechanisms at the basis of MR, concerning the number, the dendritic tree, the connections, and the excitability of PNs. However, the picture can be complicated by the possibility that some of the described alterations affect selectively discrete populations of PNs, or even discrete subregions of the same cell.

A further contribute to the complexity derives from the obvious consideration that, despite their high number, PNs are not the only determinant of cortical network properties. In fact, the interplay between PNs and GABAergic interneurons is a key element of cortical physiology (24). Early exposure to alcohol results in a change of cortical interneurons, with a significant increase of calretinin cells (7). These neurons usually co-express VIP and contact other interneurons, thus mediating disinhibition of PNs, possibly driven by feedback input from higher cortical areas (61). Therefore, the decreased intrinsic excitability of the distal apical dendrite observed in FASD (5) can be counterbalanced under certain circumstances by a relative increase of the network-mediated disinhibitory pathway.

Another puzzling issue is the apparently opposite tendency of some anatomical and electrophysiological properties in different forms of MR, as is the case for hypo- and hyperexcitability. However, this is not necessarily a contradiction, at least in terms of the functional outcome. In fact, both hypo- and hyperexcitability can equally contribute to flatten the current-frequency curve, with a reduction of the dynamic range of PNs and a consequent impairment of the ability to encode relevant information (62).

## Conflict of Interest Statement

The authors declare that the research was conducted in the absence of any commercial or financial relationships that could be construed as a potential conflict of interest.

## Supplementary Material

The Supplementary Material for this article can be found online at http://www.frontiersin.org/Journal/10.3389/fped.2014.00086/abstract
