# Supplementary material for: Alterations of Neocortical Pyramidal Neurons: Turning Points in the Genesis of Mental Retardation
Source: Front Pediatr. 2014 Aug 11;2:86. doi: 10.3389/fped.2014.00086 (PMC4127660; doi:10.3389/fped.2014.00086)
Supplement: Supplementary file 1 [file Table_1.PDF]

Supplementary Table 1. Anomalies of cortical neurons observed in experimental models of MR. For experimental models of FASD, the period of alcohol exposure is indicated in parentheses (P = postnatal day; GD = gestational day). Neurons indicated in column 1 do not represent necessarily the population selectively affected, unless the indication (select.) is present. In this case, other populations have been studied, but the alterations involve exclusively (or preferentially) the mentioned neurons. DS = Down syndrome; FX = fragile X; Hypoth = hypothyroidism; RTT = Rett syndrome.

**Affected neuron population**	**experimental model / species**	**General phenomenon**	**Observed alteration**	**Ref.**
L5 PNs (select.)	FASD / rat (P2-P6)	Increased apoptosis	Increased expression of p75 NTR	28
L5 PNs (select.)	FASD / mouse (P7)	Increased apoptosis	Increased caspase-3 immunoreactivity	27
L5 PNs (select.)	FASD / rat (P2-P6)	Increased apoptosis / lack of axon collaterals	Decreased number of cortico-cortical L5 PNs	29
L2/3 neurons (select.)	Hypoth / rat	Increased apoptosis	Increased caspase-3 immunoreactivity	30
Neocortical neurons	FX / mouse	Decreased apoptosis	Decreased caspase-3 immunoreactivity	32
L2/3 and L5 PNs	FASD / rat (P2-P6)	Altered dendritogenesis	Simplification of basal dendrites	29, 36
L2/3 PNs	DS / mouse	Altered dendritogenesis	Simplification of basal dendrites	39
L2/3 PNs	Hypoth / rat	Altered dendritogenesis	Simplification of basal and apical dendrites	42
L5 PNs	FASD / mouse (GD5-GD20)	Altered synaptogenesis	Decrease of dendritic spines	46
L5 PNs	FX / mouse	Altered synaptogenesis	Increase of dendritic spines	47
Neocortical neurons	RTT / mouse	Altered connectivity	Anomalies of axon density and orientation	50
L5 PNs	FX / mouse	Altered connectivity	Lack of connection pruning (hyperconnectivity)	51
L5 PNs	RTT / mouse	Altered connectivity	Reduced connectivity	52
